# Effects of intranasal and intramuscular dexmedetomidine in cats receiving total intravenous propofol anesthesia

**DOI:** 10.14202/vetworld.2022.1706-1713

**Published:** 2022-07-20

**Authors:** Kewvaree Hommuang, Panpicha Sattasathuchana, Naris Thengchaisri

**Affiliations:** 1 Department of Companion Animal Clinical Science, Graduate Student in Veterinary Clinical Studies, Faculty of Veterinary Medicine, Kasetsart University, Bangkok, Thailand; 2 Department of Companion Animal Clinical Sciences, Faculty of Veterinary Medicine, Kasetsart University, Bangkok, Thailand; 3 Tippimarn Veterinary Hospital, Chulabhorn Royal Academy, Nakhon Ratchasima, Thailand

**Keywords:** cats, dexmedetomidine, intranasal, propofol, sedative

## Abstract

**Background and Aim:**

The efficacy of intranasal (IN) dexmedetomidine in cats as a premedication remains elusive. Thus, this study aimed to compare the perioperative and sparing effects of IN and intramuscular (IM) dexmedetomidine administration on propofol requirements for anesthetic induction in cats.

**Materials and Methods:**

This study randomly assigned 16 cats into two groups of IN or IM dexmedetomidine at 20 μg/kg. Sedation scores and side effects were recorded at time points of 0, 5, 10, 15, and 20 min after the dexmedetomidine administration. Anesthesia was induced with intravenous (IV) 1% propofol by titrating a bolus of 2 mg every 45 s and the total dose of the administered IV propofol to achieve endotracheal intubation was recorded.

**Results:**

Cats receiving IM dexmedetomidine were significantly associated with higher sedation scores. All cats were sedated at 20 min after premedication; however, the average composite sedation scores in the IN group were significantly lower than those in the IM group during premedication. Pre-operative side effects, including vomiting, were more frequently observed in the IN group (5 cats, 62.5%) than in the IM group (3 cats, 37.5%; p < 0.05). Higher body temperature (>1°F compared to baseline) was more frequently observed in the IN group (6 cats, 75.0%) than in the IM group (1 cat, 12.5%; p < 0.05). The dosage of required propofol in the IN group was significantly higher (1.1 ± 0.3 mg/kg) than that in the IM group (0.7 ± 0.2 mg/kg; p < 0.05). The duration of general anesthesia was comparable between the groups.

**Conclusion:**

IN dexmedetomidine produces moderate sedation and cats may have side effects, including vomiting and higher body temperature. Higher sparing effects of propofol were identified in the IM group compared with the IN group. Nonetheless, IN administration of dexmedetomidine provides a noninvasive alternative to the IM route.

## Introduction

Dexmedetomidine, the active dextro-isomer of medetomidine, is a potent alpha-2 adrenoreceptor agonist that causes sedative, anxiolytic, analgesic, and sympatholytic effects [1]. Dexmedetomidine mediates its effects by stimulating the presynaptic alpha-2 adrenoreceptor, inhibiting norepinephrine release [1, 2], and stimulating postsynaptic alpha-2 adrenoreceptors on the vascular smooth muscle [3]. Dexmedetomidine is used for physical examinations of aggressive pets, minor clinical procedures, and as a pre-anesthetic medication [4–6].

Dexmedetomidine provides a strong sedative effect and significantly reduces the required dosage of anesthetic induction drugs, including propofol [7], as well as the dosage of anesthetic maintenance gas [1, 4, 7, 8]. However, dexmedetomidine can cause decreased heart rate (HR) and body temperature [1], respiratory depression [7], transient hypertension to hypotension [5], and vomiting [1, 4].

Dexmedetomidine can be injected through intramuscular (IM) or intravenous (IV) routes as recommended by the manufacturer. Intranasal (IN) dexmedetomidine provides a novel sedation method that was used in rabbits [9] and dogs [10] to facilitate clinical examination. The IN route of dexmedetomidine administration offers a clinically relevant alternative to the conventional IM route [6, 8–10]. This represents a convenient, simple, and noninvasive administration approach [8, 10]. Recently, IN dexmedetomidine has been applied in children [5, 11–13]. The drug delivery effectiveness through the IN route that targets the brain has been previously shown in humans [14, 15] and dogs [6, 8, 10]. However, the minimum necessary dose of IN dexmedetomidine for efficacy and side effects remained unknown in cats.

This study aimed to compare the sedation scores, side effects, and dose-sparing effect on propofol requirements for anesthetic induction of IN and IM dexmedetomidine administration in cats.

## Materials and Methods

### Ethical approval and informed consent

Approval was obtained from the Kasetsart University Animal Committee (ID number: ACKU64-VET-022) and from the Ethical Review Board of the Office of the National Research Council of Thailand (NRCT license U1-08175-2562). Written consent was obtained from all cat owners, and all procedures complied with the Kasetsart University Institutional Animal Care and Use Standards.

### Study period and location

The study was conducted from January 2021 to June 2021. Sixteen cats visiting the dental unit at the Kasetsart University Veterinary Teaching Hospital Bangkhen, Thailand, were enrolled in the present study.

### Animals

This study randomly selected 16 client-owned cats, including eight males and eight females, aged 2–7 years and weighing 2.5–7.0 kg, which was included in this study for dental scoring and examination based on physical examination, complete blood count, and serum biochemistry. Cats were classified into the American Society of Anesthesiologists (ASA) physical status of ASA I or II. The cats were equally and randomly assigned to either the IM or IN dexmedetomidine administration. Water and food were withheld from all the cats for 8 and 12 h, respectively, before performing the procedure.

### Study protocol

Each cat was placed in a quiet and dim room to acclimate to the environment and staff for 10 min. Then, the observer performed a physical examination before starting the experiment (T_0_). A general physical examination, including evaluation of thoracic auscultation, respiratory rate, HR, pulse palpation, mucous membrane color, capillary refill time, and body condition score, was performed by a veterinarian. The temperament of the cats was evaluated on a descriptive scale (before and after sedation) [2]. A descriptive temperament scale ranging from 0 to 2 was used [2], wherein 0 indicates that the cat does not mind physical examination, 1 indicates that the cat is scared or nervous during the physical examination, and 2 indicates that the cat is aggressive.

Cats were randomly allocated into two groups and randomly assigned to receive either IM or IN dexmedetomidine at 20 μg/kg. Each cat was restrained in sternal recumbency before drug administration. Dexmedetomidine (Dexdomitor 0.05%; Zoetis Inc., Espoo, Finland) was equally administered in the IN group by dividing the final volume into both nostrils using a 1 mL syringe without an attached needle inserted into the nostril [16]. The cat’s head was raised approximately 30° while receiving the IN dexmedetomidine. Whereas dexmedetomidine was injected into the quadriceps muscle after verifying extravascular injection in the IM group. The total volume of dexmedetomidine administration in the IN and IM groups was also recorded. The respiratory rate, HR, and rectal temperature were recorded every 10 min after T_0_. An open-label trial was used in the present study because similar premedication with the same dosage was given to the cats. Therefore, the same veterinarian that administered the drug performed the sedation scoring.

Sedation scores were assessed and recorded at time points 0 (T_0_), 5 (T_5_), 10 (T_10_), 15 (T_15_), and 20 (T_20_) min after dexmedetomidine administration. Two different sedation scoring systems were applied to assess the two administration routes of dexmedetomidine to ensure accuracy because the present study was an open-label trial. A numeric descriptive sedation scale [17] and a composite numeric rating scale [2] have been previously described in detail. Briefly, the numeric descriptive sedation scale [17] was as follows: 0, normal; 1, mild sedation; 2, moderate sedation; and 3, deep sedation. The composite numeric rating scale [2] ranged from 0 to 10, where 0 is normal and 10 is deep sedation. The composite numeric rating is the sum of the individual scores from four assessments as follows: Posture (0–4), response to clipper sounds (0–2), response to clipping (0–2), and response to restraint (0–2). This evaluation was performed by a single experienced veterinarian.

During the observation period of sedation, side effects (vomiting or higher body temperature of >1°F compared to baseline) were also recorded.

The cat was gently restrained to place an IV 24-gauge catheter in the cephalic vein for induction 20 min after drug administration. Anesthesia was induced with IV propofol (propofol 1% w/v; Troikaa pharmaceuticals Ltd., Uttarakhand, India) by titrating a bolus of 2 mg every 45 s until there was a loss of jaw tone and no/minimal gagging. The total dose of IV propofol administered to achieve endotracheal intubation was recorded. Xylocaine 10% spray (Lidocaine 10 mg/puff; AstraZeneca AB, Södertälje, Sweden) was applied to desensitize the larynx before inserting the endotracheal tube (ETT). A laryngoscope was used during ETT insertion, and its cuff was inflated to 20 cm by H_2_O using a pressure gauge. The ETT was secured with gauze. The cats were attached to a non-rebreathing system to supply 100% oxygen (250 mL/kg/min) during dental scaling after orotracheal intubation. Lactated Ringer’s solution was administered by IV at 5 mL/kg/h until extubation.

After intubation, HR, respiratory rate, oxyhemoglobin saturation (SpO_2_%), rectal temperature, palpebral reflex response, and pedal reflex response were continuously measured every 10 min using a multiparameter physiological monitor (Datex-Ohmeda CARESCAPE Multifunctional Anaesthesia Monitor; GE Healthcare Finland, Finland) and recorded at T_0_, T_10_, T_20_, T_30_, T_40_, T_50_, and T_60_. Then, 60 min after intubation, cats were given atipamezole hydrochloride (Antisedan; Zoetis Inc.) at a half-dose (100 mg/kg) concerning dexmedetomidine to facilitate recovery. Extubation was performed when the cat recovered its gag reflex. Side effects, including hyperthermia and hypersalivation, were recorded. The anesthesia duration (time elapsed from the administration of propofol to extubation) and procedure success (defined as the cats being under anesthesia that allowed dental scaling to be performed) were also recorded.

### Recovery

All cats were monitored for 1 h after extubation for upper respiratory airway discomfort, including stridor, coughing, retching, and hoarse voice. The recovery time (time elapsed from extubation to cats being capable of sternal recumbency) was recorded for each cat. After full recovery from general anesthesia, cats were returned to their owners. The owners were instructed to observe and record any abnormal signs (i.e., coughing, vomiting, restlessness, and abnormal posture) in the first 24 h at home.

### Statistical analysis

STATA12 (StataCorp, College Station, TX, USA) and GraphPad Prism version 6 (GraphPad Software, Inc., La Jolla, CA, USA) were used to estimate the required sample size using a Student’s *t*-test with a power of 80% and an alpha error of 0.05 to detect the differences in the required propofol dosage (approximately 0.5 mg/kg) between IM and IN dexmedetomidine. The average and composite sedation scores between the IM and IN groups were tested at different time points (T_0_, T_5_, T_10_, T_15_, and T_20_ after dexmedetomidine administration) using a two-way analysis of variance (ANOVA) followed by Tukey’s multiple comparison tests. The associations between the route of dexmedetomidine administration and categorical data, including side effects (vomiting and higher body temperature), body condition score, ASA status, and temperament, were determined using Fisher’s exact test. Other variables in the IM and IN groups, including propofol dosage, respiratory rate, HR, SpO_2_, and body temperature, were summarized as the mean ± standard deviation. All data were tested for normality using the Shapiro–Wilk test. A two-way ANOVA was used to compare the physiological variables between the IM and IN groups. The significance level was set at p < 0.05.

## Results

Data from the 16 analyzed cats (IN group = 8 and IM group = 8) are compared in Table-1. No statistically significant differences were identified between the IN and IM groups for age (IN: 35.4 ± 10 months, IM: 41 ± 22 months; p = 0.502), sex (IN: 4 males and 4 females, IM: 4 males and 4 females; p = 1.000), body weight (IN: 4.4 ± 1.3 kg, IM: 4.4 ± 1.0 kg; p = 0.969), body condition score (IN median BCS [range]: 3 [2–4], IM median BCS [range]: 3 [3–5]; p = 0.619), breed (IN: 7 Domestic Shorthair and 1 Persian, IM: 8 Domestic Shorthair; p = 1.000), temperament (IN median temperament [range]: 1 [0–1], IM median temperament [range]: 1 [0–2]; p = 0.282), or baseline body temperature (IN: 101.8 ± 0.6 kg, IM: 100.9 ± 1.4 °F; p = 0.119). Cats enrolled in the present study were classified according to the ASA physical status (IN median ASA [range]: 1 [1–2], IM median ASA [range]: 1 [1–2]; p = 1.000).

**Table 1:** Characteristics of cats in the IN and IM route dexmedetomidine groups.

Characteristic	IN	IM	p-value	Test
Mean age ± SD (months)	35.4 ± 10	41 ± 22	0.502	Student’s t-test
Sex (n)				
Female	4	4	1.000	Fisher’s exact test
Male	4	4		
Mean body weight ± SD (kg)	4.4 ± 1.3	4.4 ± 1.0	0.969	Student’s t-test
Median body condition score (range]	3 (2–4)	3 (3–5)	0.619	Fisher’s exact test
Median ASA status (range)	1 (1–2)	1 (1–2)	1.000	Fisher’s exact test
Breed (n)				
DSH	7	8	1.000	Fisher’s exact test
Other	1	0		
Median temperament (range)	1 (0–1)	1 (0–2)	0.282	Fisher’s exact test
Baseline body temperature	101.8 ± 0.6	100.9 ± 1.4	0.119	Student’s t-test

ASA=American Society of Anesthesiologists, DSH=Domestic shorthair, F=Female, IM=Intramuscular, IN=Intranasal, M=Male

All cats were sedated 20 min after dexmedetomidine administration via the IN or IM route.

**Figure-1:**
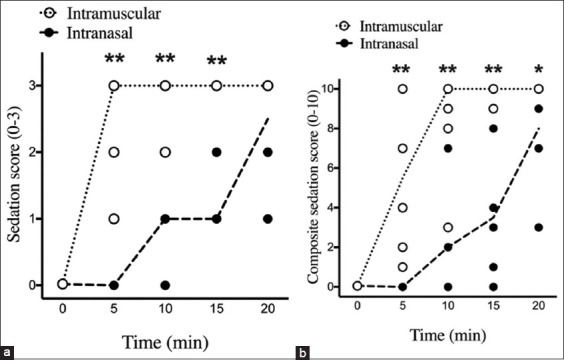
Sedation scores and composite sedation scores in cats after premedication with dexmedetomidine. The median values of each score at different time point are demonstrated in dash lines. (a) Sedation scores (0–3) in the intranasal (IN) group were significantly lower at T_5_ and T_10_ than those in the intramuscular (IM) group. (b) The IN group had significantly lower composite sedation scores at T_5_, T_10_, T_15_, and T_20_ than did the IM group. *p < 0.05 versus intranasal, **p < 0.01 versus intranasal, Two-way repeated measures analysis of variance with Tukey’s multiple comparison test.

The median and distribution and composite sedation scores are shown in Figure-1. The sedation scores in cats that received IN dexmedetomidine were significantly lower than those in the IM group at T_5_ (IN: 0 [0–2], IM: 3 [1–3]; p < 0.01), T_10_ (IN: 1 [0–2], IM: 3 [2–3]; p < 0.01), and T_15_ (IN: 1 [1–3], IM: 3 [3]; p < 0.01) (Figure-1a). The composite sedation scores in cats that received IN dexmedetomidine were significantly lower than those in the IM group at T_5_ (IN: 0 [0–4], IM: 5.5 [1–10]; p < 0.01), T_10_ (IN: 2 [0–7], IM: 10 [3–10]; p < 0.01), T_15_ (IN: 3.5 [0–9], IM: 10 [9–10]; p < 0.01), and T_20_ (IN: 8 [3–10], IM: 10 [10]; p < 0.05) (Figure-1b).

**Figure-2:**
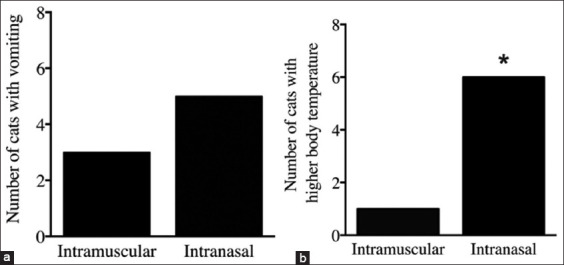
Side effects identified in cats after premedication with dexmedetomidine. (a) Number of cats with vomiting after intranasal (IN) and intramuscular (IM) dexmedetomidine administration. (b) Number of cats with higher body temperature by 1°F after IN and IM dexmedetomidine administration. *p < 0.05 versus Intramuscular, Fisher’s exact test.

After dexmedetomidine premedication, vomiting was observed in 5 (62.5%) cats in the IN group and 3 (37.5%) cats in the IM group (Figure-2a). There were significantly more cats in the IN group (6, 75.0%) than in the IM group (1, 12.5%; p < 0.05) with higher body temperature (>1°F compared to baseline; Figure-2b). We observed that high body temperature did not exceed 102.4°F.

**Figure-3:**
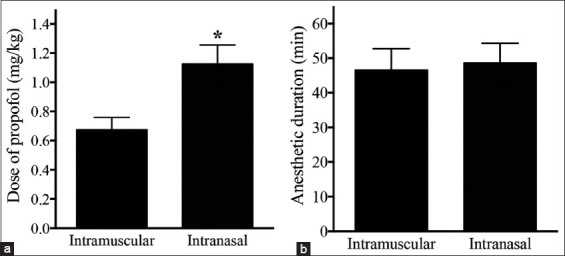
Dose of propofol required for tracheal intubation in cats premedicated with dexmedetomidine. (a): The intranasal (IN) group required a higher propofol dosage compared with the intramuscular (IM) group. (b): Anesthetic duration after propofol induction was comparable between the IN and IM groups. *p < 0.05 versus intramuscular, Student’s t-test.

**Figure-4:**
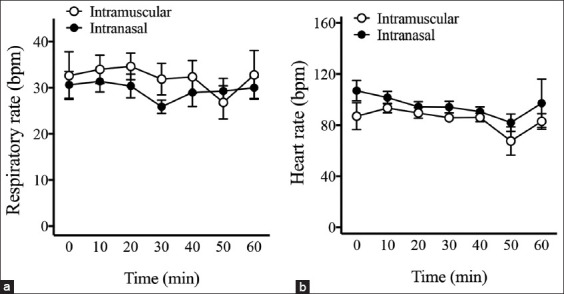
Respiratory rate and heart rate in cats receiving intravenous propofol anesthesia. (a) There was no significant difference in respiratory rate between the intranasal (IN) and intramuscular (IM) groups. (b) There was no significant difference in heart rate between the IN and IM groups. During anesthetic maintenance, respiratory rate and heart rate were not different between the two groups (p > 0.05). Two-way repeated measures analysis of variance with Tukey’s multiple comparison tests.

The dosage of propofol required for intubation after IN dexmedetomidine was significantly higher than that required after IM dexmedetomidine (IN: 1.1 ± 0.3 mg/kg, IM: 0.7 ± 0.2 mg/kg; p < 0.05) (Figure-3a). The duration of general anesthesia in cats receiving single IV propofol anesthesia was comparable between the IN and IM groups (Figure-3b). During anesthetic maintenance, the respiratory rate and HR were not significantly different between the groups (Figure-4). The respiratory rate during anesthesia was 33 ± 5 breaths/min in the IN group and 33 ± 6 breaths/min in the IM group (p > 0.05). The HR was 98 ± 15 bpm in the IN group and 88 ± 10 bpm in the IM group (p > 0.05).

## Discussion

The present study evaluated the efficacy of IN dexmedetomidine for sedation in cats and demonstrated that IN dexmedetomidine provided a lower degree of sedation compared with IM dexmedetomidine. IN dexmedetomidine decreased the amount of required propofol for endotracheal intubation but to a lesser extent compared with IM dexmedetomidine. Nonetheless, both the IN and IM groups had comparable anesthetic duration after receiving total IV propofol anesthesia.

The onset of deep sedation (descriptive sedation scores = 3; composite sedation scores = 10) in cats differs depending on the route of dexmedetomidine administration. It was greater and faster after IV injection than IM injection [7]. The onset of effect is believed to result from the drug’s high lipid solubility [18] and distribution to well-perfused tissues, such as the brain [19]. The present study revealed that most cats had deep sedation in the IN group 20 min after drug administration. Contrastingly, most cats had deep sedation in the IM group 5–10 min after drug administration. Notably, IN administration of anesthetic drugs has variable peak sedation times in different species [6, 12]. The onset of IN dexmedetomidine in humans has been reported at 45 min after administration [14, 15], whereas 6.3 ± 3.3 min in dogs [6]. The previous studies suggest that drug delivery through the nasal mucosa in dogs may be more effective than that in cats and humans. Other factors, including individual variation, drug dosage, health status, and delivery device factors, may influence drug absorption through the nasal mucosa [6, 15]. The short IN onset of sedation in animals suggests that the nose-to-brain drug delivery may rely on anatomical and physiological characteristics in the vascularization and innervation of the olfactory mucosa and bulbs that differ from those in humans [20]. Considering that this route should not be used in animals that display anatomical abnormalities is important, especially in extremely flat faces or brachycephaly skulls that decrease the nasal length, nasal epithelial surface area, and conchae structure [21].

Vomiting is a common side effect of dexmedetomidine administration in cats [2, 22]. The previous studies found that 70% of cats vomited after IM dexmedetomidine administration [23], including (67%) IV administration [22]. Dexmedetomidine and other alpha-2-adrenergic agonists cause vomiting by activating the receptors in the chemoreceptor trigger zone in cats [23, 24]. The present study revealed that vomiting occurred more in the IN group (62.5%) than in the IM group (37.5%). Contrastingly, the previous study in children [5] revealed IN dexmedetomidine as an effective for sedation and reduce the incidence of nasal irritation and post-operative nausea and vomiting compared with opioids. This result may support the suggestion that nose-to-brain delivery is an effective route for targeting the central nervous system [20]. Cats may swallow dexmedetomidine after the IN route, leading to a slower speed of sedation than the IM route. Notably, the incidence of vomiting after dexmedetomidine was not reduced by IN administration compared to the IM route.

Higher body temperature is another side effect of dexmedetomidine that has been reported in humans [25, 26]. The present study revealed increased body temperature (>1°F compared to baseline) as a significant side effect in the IN group than in the IM group after dexmedetomidine administration. Most cats in the IN group (75%) had a higher body temperature during the sedation phase. Similarly, previous studies in humans found that critically ASA III patients had elevated body temperature (≥102.2°F) shortly after receiving dexmedetomidine compared with usual care patients [25]. The clinical implications of the observed temperature effects of dexmedetomidine are unclear. In general, noradrenaline, serotonin, and dopamine are the main neurotransmitters in hypothalamic body temperature regulation [27]. Alpha-2 adrenoceptor agonists regulate the release and function of these monoamines and alter body temperature regulation. However, the mechanism of dexmedetomidine that interfere with thermoregulation is uncertain. Dexmedetomidine and other alpha-2 agonists promote hypothermia and alter the shivering threshold by the central alpha-2A adrenergic receptor action [26]. The hypothermic effect of dexmedetomidine is weakened in animals when alpha-2A adrenergic receptors are inactivated. Loss of alpha-2A receptor selectivity is associated with polymorphisms of the body, and drug interactions may increase the dexmedetomidine-induced hyperthermia occurrence [25, 26]. The current study revealed that adverse effects on increased body temperature resulting from IN dexmedetomidine administration in cats were not observed. However, IN dexmedetomidine administration should not be given to cats with a body temperature of >102.5°F. Moreover, no signs of nasal irritation after IN dexmedetomidine administration were observed in this study, similar to human results [13, 15].

Dexmedetomidine provides a strong sedative effect and significantly helps reduce the dose of induction agents, including propofol, as well as the concentration of isoflurane and sevoflurane [4, 8]. This study revealed that cats in the IN group (1.1 ± 0.3 mg/kg) required a significantly higher propofol dosage for tracheal intubation after dexmedetomidine premedication compared with those in the IM group (0.7 ± 0.2 mg/kg). For comparison, the anesthetic induction dose of propofol in cats that were not premedicated was reported as 7.3 ± 1.7 mg/kg [28]. Our study showed that IN dexmedetomidine (20 μg/kg) could reduce the required propofol dosage to achieve intubation by almost 84% (1.1 ± 0.3 mg/kg), with a 90% reduction (0.7 ± 0.2 mg/kg) for IM dexmedetomidine premedication. This marked reduction is due to central alpha-2 receptor agonists in the locus coeruleus, causing sedation, hypnosis, and synergy with GABAergic anesthetic agents [29]. However, this result may be attributed to IN premedication benefits, which produce a sedative effect in cats and a lower propofol requirement for the anesthesia induction than that in less-sedated cats. Similarly, a study conducted by Robinson and Borer-Weir [30], showed that cats with a high level of sedation required less propofol than those not premedicated for anesthesia induction. During the induction phase, adverse effects of propofol administration, such as apnea or excitatory phenomena, were not observed.

During the anesthetic maintenance, the respiratory and HR after receiving IV propofol anesthesia did not differ between the two groups and likely reflected the dexmedetomidine effects (HR = 98 ± 15 bpm in the IN group and HR = 88 ± 10 bpm in the IM group). Dexmedetomidine is known to cause bradycardia by vasopressor action that increases arterial and pulmonary pressures [5]. In addition, this drug decreases sympathetic nervous system activity within the central nervous system, thereby decreasing both GABAergic and glycinergic inhibitory input into the cardiac vagal neurons, which may worsen bradycardia [31]. In general, cats with bradycardia had HR that was profoundly below 100 bpm, which agrees with our observations. Furthermore, our study showed that anesthetic duration was not significantly different between the two groups (IN: 48 ± 15 min, IM: 46 ± 17 min). Further, atipamezole was applied at the end of the study. The recovery time may be prolonged without a reversal agent. The pharmacokinetics and relative bioavailability of the IN versus IM route may also be different since a higher propofol dosage was required for the IN group’s anesthesia induction.

IN administration is an alternative route to sedating and placing chemical restraints on cats. Dexmedetomidine is a drug formulation that provides deep sedation and analgesia and is used for premedication before the induction and maintenance of general anesthesia in cats. Our trial revealed that IN dexmedetomidine administration was easy to perform, noninvasive, and well-tolerated, although it caused snorting or a sneezing reaction in the cats after administration. However, IN administration is an alternative route that possibly reduces the stress and pain caused by IM dexmedetomidine administration. Nasal drug delivery is an effective and noninvasive alternative method to the IM route for dexmedetomidine sedation in cats.

This study had several limitations, including the small number of cats tested, the dose titration of dexmedetomidine by IN route, and the lack of recorded blood pressure during anesthesia. In addition, a pharmacokinetic study should be conducted to determine the bioavailability of IN sedation compared with other routes of administration.

## Conclusion

Based on our findings, IM and IN dexmedetomidine administration provided a good sedation level that is sufficient to reduce the amount of anesthesia-inducing agents. However, the sedation levels in cats that received IN dexmedetomidine were significantly lower than those in the IM dexmedetomidine during premedication. Moreover, cats may have some side effects, including vomiting and higher body temperature, after IN dexmedetomidine. Higher sparing effects of propofol were identified in the IM group compared with the IN group. Nonetheless, IN dexmedetomidine administration is a potential noninvasive alternative to IM administration.

## Authors’ Contributions

KH, PS, and NT: Conceptualization. KH and NT: Conducted experiments. KH, PS, and NT: Methodology. KH, PS, and NT: Data analysis. KH: Drafted the manuscript. KH, PS, and NT: Reviewed and edited the manuscript. All authors have read and approved the final version of the manuscript.
